# A retrospective study: management of Rhizobium radiobacter-associated bloodstream infections in pediatric hematology and oncology patients

**DOI:** 10.1590/1806-9282.20241944

**Published:** 2025-07-07

**Authors:** Onur Tekeli, Tuğce Tural Kara, Hafize Selma Çetin, Hatice Burcu Çağlar Kızıl, Ayşe Kübra Açık, Funda Tayfun Küpesiz, Meral Dilara Oğünç

**Affiliations:** 1 Akdeniz University, Faculty of Medicine, Department of Pediatric Infectious Diseases – Antalya, Turkey.; 2 Akdeniz University, Faculty of Medicine, Department of Pediatric Hematology and Oncology – Antalya, Turkey.; 3 Akdeniz University, Faculty of Medicine, Department of Clinical Microbiology – Antalya, Turkey.

**Keywords:** Rhizobium radiobacter, Bloodstream infection, Central venous catheters

## Abstract

**OBJECTIVE:**

*Rhizobium* spp. strains (formerly classified as *Agrobacterium* based on 16S rDNA analysis) are aerobic, motile, oxidase-positive, non-spore-forming gram-negative bacilli. Clinical manifestations associated with *Rhizobium radiobacter* infections vary widely, with bacteremia typically linked to intravenous catheter use.

**METHODS:**

This study analyzes the clinical and laboratory characteristics of 11 pediatric hematology and oncology patients at a tertiary referral hospital who had *Rhizobium radiobacter*-associated bloodstream infections over a 3-month period.

**RESULTS:**

Nine of the patients were male, and hematologic malignancy was the primary diagnosis in seven patients. Common risk factors included hospitalization within the last month, chemotherapy treatment, and the presence of an indwelling central venous catheter in all cases. *Rhizobium radiobacter* growth was detected an average of 13.5±16.6 days (range 1–54 days) after hospital admission, with 36% of patients being neutropenic at the time of culture positivity. *Rhizobium radiobacter* growth was detected in both catheter and peripheral blood cultures in six patients, whereas the microorganism was isolated exclusively from catheter cultures in four patients. Ten patients received antibiotic lock therapy, predominantly with amikacin (90%). Systemic antibiotic treatment involved a combination of beta-lactam and aminoglycoside antibiotics in 10 patients. Culture sterility was achieved in nine patients by the third day of antibiotic treatment. There were no cases of catheter extraction, thrombophlebitis, cardiac vegetation, reproductive complications, or mortality.

**CONCLUSION:**

In this first pediatric case series utilizing antibiotic lock therapy, effective treatment, strict isolation, and surveillance screenings led to favorable clinical outcomes for *Rhizobium radiobacter*-associated bloodstream infections in children with hematologic and oncologic conditions.

## INTRODUCTION

Strains of *Rhizobium* species (formerly Agrobacterium, reclassified based on 16S rDNA analysis) are aerobic, motile, oxidase-positive, and non-spore-forming gram-negative bacilli. The genus Agrobacterium encompasses environmental opportunistic human pathogens found in soil and plants, with most infections reported in immunocompromised hosts such as oncology patients, the elderly, and neonates. *Agrobacterium radiobacter* lacks the tumorigenic Ti plasmid present in *Agrobacterium tumefaciens*, making it non-phytopathogenic. Central venous catheter (CVC)-associated bloodstream infection (BSI) is the most common clinical manifestation and can occasionally be preceded by soil contact^1,2^. However, *A. radiobacter* is increasingly associated with human infections and was first reported in 1980 in a case of prosthetic valve endocarditis^3^. Since 2003, *Rhizobium radiobacter* has replaced *Agrobacterium radiobacter* in nomenclature. Of the *Rhizobium* species (e.g., *R. radiobacter*, *R. rhizogenes*, *R. rubi*, *R. undicola*, and *R. vitis*), *R. radiobacter* is the most common cause of human disease, associated with systemic conditions like peritonitis, urinary tract infections, myositis, and infections of intravascular devices in immunocompromised patients^4^. Most patients with *R. radiobacter* infection have shown a good response to antibiotic treatment. Moreover, an important factor in the therapy is the removal of foreign material, as patients with persistent infection or those who initially respond to treatment are at risk of relapse until the foreign body is removed. The prognosis for *R. radiobacter* infection is generally favorable^5,6^.

In this study, the clinical and laboratory features and outcomes of *R. radiobacter-*associated BSI in pediatric patients hospitalized in the pediatric hematology and oncology clinics within a 3-month period.

## PATIENTS AND METHODS

The presence of fever, chills, tachycardia, and hypotension, either alone or in combination, with the growth of microorganisms in blood cultures, is considered diagnostic of a bloodstream infection. Catheter-related BSI is defined by the growth of microbes in a blood sample drawn from a catheter hub at least 2 h before microbial growth is detected in a peripheral vein blood sample^7^.

The clinical and laboratory findings, such as age, gender, primary diagnoses, and comorbid disease of patients with *R. radiobacter*-associated BSI between May and July 2023 are analyzed retrospectively.

In addition, clinical and laboratory findings, blood culture growth times, and antimicrobial susceptibility test results were reviewed from electronic patient files. We analyzed antibiotic treatment combinations (both systemic and lock therapy) and outcomes, as well as complications and 30-day mortality.

Blood samples were collected using BD BACTEC Peds Plus/F medium, and *R. radiobacter* bacteria were identified using matrix-assisted laser desorption/ionization time-of-flight mass spectrometry (MALDI-TOF MS) on a Bruker Biotyper device. Antimicrobial susceptibility tests were conducted using automated methods on a BD Phoenix 100 system (Becton Dickinson, Sparks, MD, Germany) and manual E-test.

The data were entered and analyzed using descriptive statistical analysis with the Statistical Package for the Social Sciences (SPSS) program version 26. Descriptive statistical analyses were performed. There was no control group or comparator. As this was a retrospective data review, informed consent was not required from the patients. Prior to commencing the study, ethical approval was obtained from the Medical Scientific Research Ethics Committee of Akdeniz University, in accordance with national guidelines (TBAEK-314), dated April 25, 2024.

## RESULTS

### Demographic and clinical findings

The mean age of the patients was 9.2±4.2 years (range: 4–16), and nine of them were male. The primary diagnosis was hematologic malignancy in seven patients and solid tumor in four patients. Comorbidities were present in 45.5% of the patients. As comorbidities, case 1 presented with jejunostomy and invasive pulmonary aspergillosis, case 3 with osteoporosis, case 4 with hematopoietic stem cell transplantation and invasive pulmonary aspergillosis, case 5 with beta thalassemia minor, and case 6 with hepatitis B carrier status. All patients had been hospitalized within the last month, received chemotherapy, and had an indwelling central venous catheter, specifically a tunneled central venous catheter (Hickman Type) in 10 cases. Fever was the sole symptom at the time of culture, with one patient also experiencing oral mucositis.

### Laboratory and microbiological findings

*R. radiobacter* was isolated from blood culture on average 13.5±16.6 days (range: 1–54 days) after hospitalization, with 36% of cases being neutropenic at this time. Therefore, all infections were considered healthcare-associated infections. Patients with growth only in catheter blood cultures were not neutropenic at the time of culture growth. Laboratory findings included a mean leukocyte count of 3,445.5±2,991.2/mm³ (range: 30–9,570), an absolute neutrophil count of 2,329.1±1,959.3/mm³ (range: 0–6,580), a C-reactive protein level of 33.9±45.9 mg/L (range: 0.6–121), and a procalcitonin level of 1.67±2.3 ng/mL (range: 0.17–7.7). Detailed clinical, laboratory, and prognostic characteristics of the patients are summarized in Table 1, and the antimicrobial susceptibility results are shown in Table 2.

**Table 1:** Demographic, clinical, laboratory, and treatment characteristics of patients with *Rhizobium radiobacter* bacteremia.

Case	Age, years	Gender	Primary diagnosis	Neutropenia	Bacteremia	Antibiotic therapy		Catheter removal	Mortality
						Systemic	Lock		
1	14	M	BL	No	P+C	TZP, AMI	AMI	No	No
2	14	M	Osteosarcoma	No	P+C	MEM, GEN	GEN	No	No
3	6	F	NHL	Yes	P+C	CEF, AMI	AMI	No	No
4	7	M	Relapsed ALL (B-cell)	Yes	P	MEM, CT	–	No	No
5	16	M	Relapsed LL (T-cell)	No	C	CEF, AMI	AMI	No	No
6	13	M	BL	No	C	CEF, AMI	AMI	No	No
7	6	F	B-cell LL	No	C	TZP, AMI	AMI	No	No
8	7	M	ALL (B-cell IR)	No	P+C	CRO, AMI	AMI	No	No
9	8	M	ALL (B-cell IR)	No	C	CRO, AMI	AMI	No	No
10	4	M	ALL (B-cell HR)	No	P+C	CRO, AMI	AMI	No	No
11	6	M	ALL (T-cell)	Yes	P+C	TZP, AMI	AMI	No	No

M: male; F: female; BL: Burkitt lymphoma; NHL: non-Hodgkin lymphoma; ALL: acute lymphoblastic leukemia; LL: lymphoblastic lymphoma; IR: intermediate risk; HR: high risk; P: peripheral; C: catheter; P+C: peripheral and catheter; TZP: piperacillin-tazobactam; AMI: amikacin; MEM: meropenem; GEN: gentamicin; CEF: cefepime; CRO: ceftriaxone; CT: colistin.

**Table 2:** Antimicrobial susceptibility test results of *Rhizobium radiobacter* isolates with BD Phoenix 100 (Becton Dickinson, Sparks, MD, Germany) and E-test.

	BD Phoenix 100	E-test
Antibiyotik	MIC_50_ (mcg/mL)	MIC_50_ (mcg/mL)
AM (ampicillin)	8	
AMX (amoxicillin)	8	
AN (aminosidin)	≤4	
AXC (amoxicillin-clavunate)	16/2	
CAZ (ceftazidime)	16	256
CFM (cefixime)	>2	
CIP (ciprofloxacin)	≤0.0625	
CL (cephalexin)	≤1	
CT (colistin)	>2/4	
CTX (cefotaxime)	>4	32
CXM (cefuroxime)	>16	
ETP (ertapenem)	≤0.25	
FF (fosfomycin)	>64	
GEN (gentamicin)	2	2
IPM (imipenem)	≤0.25	
LVX (levofloxacin)	≤0.5	
MEM (meropenem)	≤0.125	2
NN (tobramycin)	8	
OFX (ofloxacin)	≤0.5	
SXA (ampicillin-sulbactam)	≤1/8	
SXT (trimethoprim-sulfamethoxazole)	≤1/19	
TGC (tigecycline)	≤1	
TZP (piperacillin-tazobactam)	8/4	

MIC_50_ (mcg/mL): minimum inhibitory concentrations (MIC) that inhibited 50% of the tested microorganisms.

### Treatment outcomes

*R. radiobacter* was isolated from both the catheter and peripheral blood cultures in six patients and exclusively from the catheter blood culture in four patients. Ten patients received catheter lock treatment, with 90% treated with amikacin. Systemic antibiotic treatments, comprising a combination of beta-lactam and aminoglycoside antibiotics, were administered to 10 patients. Culture sterility was achieved in nine patients by the third day of antibiotic treatment. None of the patients required catheter extraction, developed thrombophlebitis, cardiac vegetation, growth-related complications, or mortality.

## DISCUSSION

In patients with long-term central venous catheter use, common causative agents of catheter-related bloodstream infection (CRBSI) include coagulase-negative Staphylococcus, gram-negative enteric bacilli, *Staphylococcus aureus*, and *Pseudomonas aeruginosa* ^7^. Although CRBSI caused by *R. radiobacter* is uncommon, it has been documented in the literature often associated with immunosuppression and CVC use.

By 1977, a total of 37 *R. radiobacter*-associated growths were identified in clinical isolate cultures, and these were considered as contamination or colonization^8,9^. From the first case in 1980, prosthetic valve endocarditis caused by *R. radiobacter* ^3^, to 2024, "*R. radiobacter*"-related infections were screened in PubMed. A total of 76 reports were found, of which three were pseudo-outbreaks and one reported epidemiological findings. In the remaining 72 reports, 111 *R. radiobacter*-associated infections were reported. The search was narrowed using the terms "human," "English language only," and "full text availability." A total of 55 publications that met the criteria were analyzed, and CRBSI cases caused by *R. radiobacter* in children are detailed in Table 3 ^5,6,10–18^. The distribution and types of infections according to age groups are as follows: Four cases of bacteremia were reported in neonates (four publications), 16 cases of catheter-related bacteremia and one case of peritonitis were reported in children (12 publications), and in adults (40 publications), there were catheter-related bacteremia (n=20), bacteremia (n=12), keratitis (n=15), endophthalmitis (n=7), peritonitis (n=6), pneumonia (n=4), endocarditis (n=3), and one case each of cerebral abscess, skin abscess, and spondylodiscitis.

**Table 3:** Literature review of pediatric patients with catheter-related bloodstream infection caused by *R. radiobacter.*

Reference number/publication year	Age (year)/gender	Primary disease	Antibiotic therapy		Catheter removal/source of infection
			Systemic	Lock	
This report/2024 For details, see Table 1					
10/2012	1.5/F	ALL	IPM	No	No/CAI
11/2010	7/M	ALL	CRO	Yes-GEN	No/CAI
12/2010	0.5/F	SCID, HSCT	CEF, AMI	No	No/CAI
13/2003	6/F	VAHS	TCC, GEN	No	Yes/CAI
	6/NS	ALL	CAZ, GEN	NS	No/NS
	10/M	ALL	TCC, GEN	No	Yes/CAI
	2/NS	NBL	TCC, GEN	NS	Yes/NS
	3/NS	ITP	TCC, GEN	NS	No/NS
14/1997	NS/NS	NBL	NS	NS	Yes/CAI
5/1993	5/M	ALL	CAZ, GEN	No	No/CAI
	4/M	ALL	CXM, IPM	No	Yes/CAI
15/1993	3/M	CNS PNET	TCC, GEN	No	NS/CAI
6/1993	4/M	RMS	CAZ	No	No/HAI
	0.6/F	SCID	NS	No	Yes/HAI
16/1991	6/NS	AIDS	CRO	No	No/CAI
17/1989	16/M	AA	MZ, OX, GEN	No	Yes/HAI
18/1989	1.2/M	SBS	TIC, IPM	No	Yes/HAI

NS: not specified; SCID: severe combined immunodeficiency; HSCT: hematopoietic stem-cell transplantation; VAHS: viral-associated hemophagocytic syndrome; NBL: neuroblastoma; ITP: chronic severe trombocytopenia; CNS PNET: central nervous system primitive neuroectodermal tumor; RMS: rhabdomyosarcoma; AIDS: acquired immunodeficiency syndrome; AA: aplastic anemia; SBS: short bowel syndrome; HAI: healthcare associated infection; CAI: community acquired infection; IPM: imipenem; TCC: ticarcilline-clavulanic acid; CAZ: ceftazidime; CXM: cefuroxime; MZ: mezlocillin; OX: oxacillin; TIC: ticarcillin; ALL: acute lymphoblastic leukemia; CRO: ceftriaxone; CEF: cefepime; AMI: amikacin; GEN: gentamicin.

The last two adult CRBSIs caused by *R. radiobacter* reported in the literature were reviewed. In Italy, in 2017, a 77-year-old man who was followed with an indwelling tunneled hemodialysis catheter due to renal neoplasia was successfully treated with systemic and lock levofloxacin therapy for CRBSI^19^. In 2008, a 51-year-old male in India was admitted with acute coronary syndrome and recurrent melena. He was diagnosed with an *R. radiobacter* infection in a temporary central venous catheter and was treated with tigecycline and imipenem antibiotics, without the need for catheter removal^20^.

Consistent with the literature findings, our patients had a history of prolonged hospitalization, chemotherapy, and immunosuppressive drug use, with all of them having indwelling CVC. CRBSI cases caused by *R. radiobacter* were effectively managed with systemic and lock antibiotic therapy. Antibiotic MIC_50_ values were summarized in Table 2. However, due to the lack of standardized antibiogram results for *R. radiobacter* according to the European Committee on Antimicrobial Susceptibility Testing, a manual E-test (Table 2) was performed by the microbiology laboratory. Cefotaxime, meropenem, and gentamicin were considered susceptible, whereas ceftazidime was considered resistant.

Despite recent chemotherapy and regular immunosuppressive medication in our patients, initial broad-spectrum antibiotic treatment was initiated based on previous culture results, accompanying febrile neutropenia, deterioration in general condition, and comorbidities. Catheter lock treatment resulted in culture sterility by the third day in 90% of patients and by the 14th day in the remainder, with no cases requiring catheter removal. None of our patients had any host factors that would limit catheter lock therapy, such as severe sepsis, hemodynamic instability, or additional CRBSI caused by *S. aureus*, *P. aeruginosa*, or *Candida* spp. Regarding potential side effects of catheter lock therapy, no fungal superinfection, anticoagulant or antibiotic toxicity, or development of antibiotic resistance was observed during the 30-day follow-up, and following catheter lock therapy, the catheters were re-accessed with no catheter-related bloodstream infections caused by *R. radiobacter* observed, as the patients remained clinically stable throughout the follow-up period.

*R. radiobacter* sepsis was reported in a 27-year-old female patient with known sickle cell anemia and Munchausen's syndrome in the United States, self-administered by intravenous injection of feces and dirt^21^. In Australia, a 44-year-old man who was an intravenous drug user and stored drug paraphernalia in plant beds presented with arterial thrombus and cardiac vegetation caused by *R. radioabcter* ^22^. Although there are publications^23,24^ reporting that *R. radiobacter*-associated CRBSI can develop following environmental contamination and soil contact, no such history was found in our cases.

In a study conducted by Stern et al.^25^ in June 2023 in the United States, *R. radiobacter* growth was observed in the tissue cultures of six patients. The reason for the growth in patients without clinical and laboratory findings of infection was errant laboratory tissue processing with contaminated, nonsterile saline. In our study, the infection control committee was consulted to investigate the infection source. Environmental cultures (from shared saline, disinfectants, and heparin) were obtained in cases without soil contact to investigate possible pseudo-epidemic scenarios and manage treatment sources, but no epidemic source was identified.

Our study has a limitation in that it was conducted with an immunosuppressive and limited patient group, and pulsed-field gel electrophoresis and next-generation MALDI-TOF MS-based whole-exon sequencing were not technically available to investigate genetic similarity among *R. radiobacter* isolates.

CRBSI caused by *R. radiobacter* is a rare disease in pediatric patients. Catheter removal related to *R. radiobacter* occurred in 50% of CRBSI cases in children. This report is important as it shows that CRBSI caused by *R. radiobacter* can be treated with systemic and lock antibiotic therapy without the need for catheter removal. In conclusion, favorable clinical outcomes may be achieved through appropriate treatment, strict isolation measures, and surveillance screening for *R. radiobacter*-associated BSI.

## Data Availability

The datasets generated and/or analyzed during the current study are available from the corresponding author upon reasonable request.
